# Suppression of Tumor Angiogenesis by Nonsteroidal Anti-Inflammatory Drugs: A New Function for Old Drugs

**DOI:** 10.1100/tsw.2001.396

**Published:** 2001-12-01

**Authors:** Curzio Raegg, Olivier Dormond

**Affiliations:** Laboratory of the Centre Pluridisciplinaire d'Oncologie, University of Lausanne Medical School, Swiss Institute for Experimental Cancer Research, Epalinges, Switzerland

**Keywords:** cancer, integrins, angiogenesis, NSAIDs, endothelial cells, Rho-family GTPases

## Abstract

There is solid epidemiological evidence demonstrating that the regular use of nonsteroidal anti-inflammatory drugs (NSAIDs) reduces the risk of developing colorectal cancer, and to a lesser extent gastric and esophageal cancers[1]. Importantly, NSAIDs suppress colon polyp formation and progression in patients diagnosed with familial adenomatous polyposis coli (APC)[2]. In many animal studies, NSAIDs have been shown to prevent tumor formation and slow tumor progression, thus confirming and extending the clinical observations[3,4,5]. Recent findings have demonstrated that NSAIDs inhibit angiogenesis, suggesting that the tumor suppressive activity of these drugs may be due, at least in part, to their ability to inhibit tumor angiogenesis[6]. The study of the mechanism by which NSAIDs suppress tumor angiogenesis, is matter of intense research.

In a recent paper published in *Nature Medicine*[7], we reported that inhibition of COX-2 by the nonselective cyclooxygenase (COX) inhibitor indomethacin or the selective COX-2 antagonist NS-398 blocked FGF-2-induced angiogenesis in mice. *In vitro*, indomethacin and NS-398 suppressed endothelial cell spreading and migration mediated by integrin $\alpha V\beta 3$, an adhesion receptor critically involved in promoting tumor angiogenesis[8]. This effect was associated with the suppression of $\alpha V\beta 3$-dependent activation of Cdc42 and Rac, two members of the Rho family of GTPases that regulate cytoskeletal organization and cell migration. Expression of a constitutive active form of Rac prevented NS-398–induced suppression of endothelial cell spreading and migration *in vitro* and reversed the inhibition of angiogenesis caused by NS-398 *in vivo*. Taken together, these results implicate that inhibition of $\alpha V\beta 3$-mediated Rac activation is an important mechanism by which NSAIDs suppress endothelial cell migration and angiogenesis.

COX-1 and COX-2 are the rate-limiting enzymes in the conversion of arachidonic acid into prostaglandins (e.g., $PGE_2$, $PGF_{2\alpha}$, $PGD_2$, $PGI_2$) and thromboxans[9]. COX-1 is constitutively expressed in most tissues and plays an important role in tissue homeostasis. In contrast, COX-2 is absent from most normal tissues and its expression is up-regulated in inflammation[10] and in many cancers, including colon, breast, prostate, and skin[11].

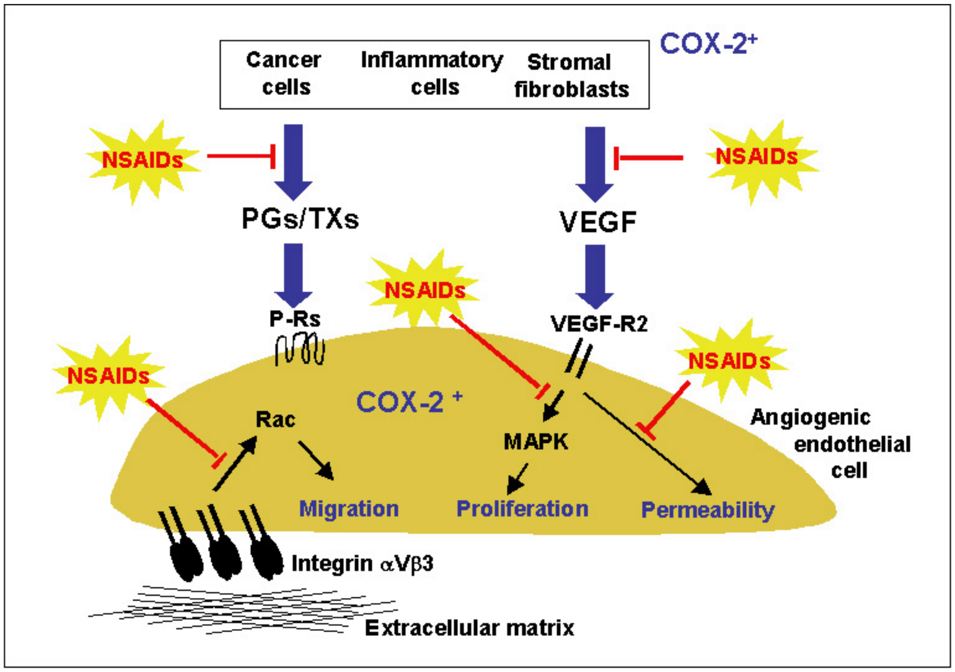

**FIGURE 1.** Schematic representation of the effects of NSAIDs on angiogenic endothelial cells. Cancer cells, stromal fibroblasts, infiltrating inflammatory cells and angiogenic endothelial cells up-regulate COX-2 resulting in increased production of prostaglandins and thromboxane (PG/TX). PG/TX bind to prostanoid receptors and regulate signaling from the VEGF receptor 2 (VEGF-R2) and integrin αVβ3. NSAIDs inhibit PG/TX and VEGF production, suppress endothelial cell proliferation and vascular permeability in response to VEGF and inhibit αVβ3 integrin-dependent Rac activation and endothelial cell migration.

Within the tumor microenvironment, COX-2 is expressed by cancer cells, stromal fibroblasts, tumor infiltrating inflammatory cells, and angiogenic endothelial cells[11]. Consistent with a role of COX-2 in tumor progression, inhibition of COX-2 by NSAIDs reduced the growth of many experimental tumors. For example, topical application of diclofenac, a nonselective NSAID, retarded Colon-26 tumor growth in mice and this effect was associated with suppressed tumor angiogenesis[12]. Subsequently it was shown that COX-2 antagonists inhibited growth factor–induced angiogenesis. In a rat model of angiogenesis the COX-2 inhibitor celecoxib, blocked corneal blood vessel formation while SC-560, a specific COX-1 inhibitor had no effect[11].

Previous reports demonstrated that COX-2 regulates the expression of Vascular Endothelial Growth Factor (VEGF) as well as the biological response of endothelial cell response to VEGF. Mice lacking the COX-2 gene had deficient production of VEGF by fibroblasts and treatment of wild-type fibroblasts with a selective COX-2 inhibitor suppressed VEGF production[13]. Overexpression of COX-2 in colon cancer cells induced expression of VEGF and other angiogenic factors and this effect was inhibited by the COX-2–specific antagonist NS-398[6]. Consistent with these findings, prostaglandins enhanced VEGF production in many different cells[14]. COX-2 is also involved in the regulation of VEGF-induced vascular permeability and endothelial cell proliferation. Indomethacin blocked increased vascular permeability in response to VEGF while administration of prostaglandin $I_2$ increased vascular permeability[15]. Inhibition of COX-2 by NS-398 prevented VEGF-mediated MAPK activation and endothelial cell proliferation[16]. Taken together, these reports demonstrate that NSAIDs suppress tumor angiogenesis by inhibiting at least three different events of the angiogenic cascade: down-regulation of the production of angiogenic factors, inhibition of the endothelial cell response to VEGF, and suppression of integrin αVβ3-dependent endothelial cell migration.

Where do we go from here? First, we should further investigate the molecular effects of NSAIDs on endothelial cells. For example, we know little about the intracellular events responsible for the inhibition of VEGF-induced MAPK activation and the suppression of αVβ3-mediated cdc42 and Rac activation. Second, we should evaluate the potential therapeutic effects of this drug class as antiangiogenic agents in human cancer. Choosing the right patients, therapy regimens, and endpoints will be essential for the success of these trials.

**Choice of the type of cancer and stage.** Colorectal cancer is the prime choice, since we already know that NSAIDs protect against this cancer. It may also be important to include patients at different tumor stages, for example disease-free patients at high risk for recurrence (e.g., stage III colon cancer) and patients with advanced colon cancer (e.g., liver metastasis). Indeed we know from preclinical studies that antiangiogenic drugs given at different stages of tumor progression produce distinct efficacy profiles[17].

**Therapy combination and schedule.** Since it is unlikely that NSAIDs will have any therapeutic effects when given alone, they should be tested in combination with chemotherapy or radiotherapy. This concept is supported by experimental data[18,19]. In addition, it will be important to compare the effects of synchrone vs. alternate administration in relationship to chemotherapy or radiotherapy.

**Evaluation of NSAIDs anticancer effects.** This is a critical point common to all clinical trials involving antiangiogenic drugs. Since the antitumor effect of antiangiogenic drugs is indirect, it will be important to assess the effects on the tumor vasculature independently of the overall antitumor response. Imaging of the tumor vasculature by Computer Assisted Tomography (CAT scan), Magnetic Resonance Imaging (MRI), Positron Emission Tomography (PET), and Power-Doppler[20-22] allows to detect effects on tumor vessels. These techniques, however, have some limitations and the identification of sensitive and reliable biological and surrogate markers of angiogenesis will be instrumental to the clinical evaluation of antiangiogenic drugs, including NSAIDs.
